# The effectiveness of non-invasive brain stimulation in enhancing lower extremity function in children with spastic cerebral palsy: Protocol for a systematic review and meta-analysis

**DOI:** 10.1016/j.mex.2024.103141

**Published:** 2024-12-31

**Authors:** Clàudia Arumí-Trujillo, Francisco José Verdejo-Amengual, Oriol Martínez-Navarro, Jord J.T. Vink, Fran Valenzuela-Pascual

**Affiliations:** a Faculty of Nursing and Physiotherapy, Universidad de Lleida, Roig 2, 25198 Lleida, Montserrat, España; b Group of Studies on Society, Health, Education and Culture (GESEC), Universidad de Lleida, Pl. de Víctor Siurana 1, 25003 Lleida, España; c Research Group of Health Care (GReCS), Lleida Biomedical Research Institute's Dr. Pifarre Foundation (IRB Lleida), Avda Alcalde Rovira Roure 80, 25198 Lleida, España; d Translational Neuroimaging Group, Center for Image Sciences, University Medical Center Utrecht and Utrecht University, the Netherlands; e Center of Excellence in Rehabilitation Medicine, University Medical Center Utrecht Brain Center, University Medical Center Utrecht, Utrecht University and De Hoogstraat Rehabilitation, the Netherlands

**Keywords:** Transcranial Magnetic Stimulation, Transcranial Direct Current Stimulation, Cerebral Palsy, cortical excitability, Motor skills, Muscle spasticity, A protocol for a systematic review and meta-analysis to evaluate the effectiveness of non-invasive brain stimulation in enhancing lower extremity function in children with spastic cerebral palsy

## Abstract

Non-invasive brain stimulation (NIBS^1^) techniques have emerged as a promising non-pharmacological adjunct to neurorehabilitation. Children with Cerebral Palsy (CP^2^) exhibit altered cortical excitability, and while CP remains incurable, physiotherapy combined with other interventions is essential for managing motor dysfunction. Although some studies have examined NIBS using various stimulation parameters, there is limited evidence regarding its effects on the lower extremities and optimal administration protocols. This review aims to evaluate the effectiveness of NIBS techniques, such as transcranial direct current stimulation (tDCS^3^) and repetitive transcranial magnetic stimulation (rTMS^4^), for the treatment of motor function in spastic cerebral palsy, specifically in the lower extremity. A systematic search will be conducted in databases including MEDLINE, CINAHL Plus, EMBASE, Scopus, ISI Web of Science, and the Cochrane Central Register of Controlled Trials. The search strategy will follow the PICO framework (Participants, Intervention, Comparison, Outcomes), focusing on randomized controlled trials (RCTs). Two independent reviewers will manage screening, selection, data extraction, risk of bias assessment, and grading of evidence. This review will provide key insights into the effectiveness of NIBS for lower-extremity function in children with spastic CP, guiding future research and clinical applications.

## Graphical abstract

**Figure:**
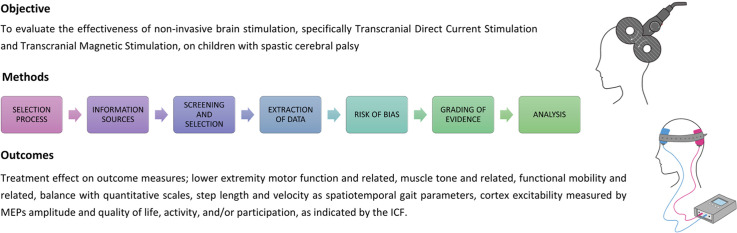

Specifications table

**Table:** 

Subject area:	Medicine and Dentistry
More specific subject area:	Neurorehabilitation
Name of your protocol:	A Protocol for a Systematic Review and Meta-analysis
Reagents/tools:	Not applicable
Experimental design:	Systematic review study
Trial registration:	PROSPERO CRD42024602482
Ethics:	This study will not involve humans.
Value of the Protocol:	• This review protocol may help develop evidence-based strategies to optimize the use of non-invasive brain stimulation techniques, potentially improving outcomes for children with cerebral palsy. • Establishing new, reliable estimates for the effectiveness of various NIBS techniques in managing motor impairments in children with cerebral palsy is essential for informed clinical decision-making. • This protocol could enhance our understanding of the impact of NIBS interventions on motor function improvement and related outcomes, providing valuable insights for future healthcare interventions and clinical guidelines.

## Background

Cerebral palsy (CP) is not a distinct disease classification, but rather an umbrella term encompassing aetiologically diverse symptoms [1]. Cerebral Palsy is defined as a group of lifelong motor disorders resulting from non-progressive brain disturbances occurring during fetal or early infant development. CP primarily impairs movement and posture, limiting activity, and is frequently associated with sensory, perceptual, cognitive, communicative, and behavioural disturbances, as well as epilepsy and secondary musculoskeletal complications [2,3]. Muscle tone changes due to brain injury, particularly hypertonicity, is the main motor disorder in 80-90 % of children with CP. These alterations can hinder coordination, strength, and selective motor control [4,5]. Damage to pathways interacting with the corticospinal tract, like the reticulospinal tract, impairs inhibitory control, increasing muscle tone. In children who suffer from an early brain abnormality, spasticity results from disrupted supraspinal input and impaired motor maturation, leading to a loss of descending inhibition and heightened excitability of gamma and alpha neurons [6].

Spasticity management typically focuses on medication, such as baclofen or diazepam, among others. However, these drugs primarily target muscle relaxation and often come with side effects. First-line treatments for spasticity include physiotherapy, occupational therapy, botulinum toxin injections, selective dorsal rhizotomy, and intrathecal baclofen [7,8]. Rehabilitation is grounded in the concept of neuroplasticity, which is a key factor for learning and memory, as well as a foundation for adaptation [9].

The non-progressive neurological disturbances in the infant brain leads to significant mobility limitations, which can negatively impact CP children's quality of life, social interactions, and overall well-being. By addressing the underlying causes of lower extremity dysfunction, novel therapeutic approaches, such as neurorestorative therapies, can potentially reduce disability, enhance independence, and improve long-term outcomes [10,11].

The use of NIBS techniques in the field of neurorehabilitation has gained momentum since the mid-2000s. It has turned into a potential technical adjuvant to neurorehabilitation treatments. By employing electrical and magnetically-induced currents, the brain is stimulated through the scalp, temporarily exciting or inhibiting activity in targeted brain areas [12]. Recently, new research lines in the NIBS field have focused on paediatric neurological diseases as a potential candidate to manage spasticity and motor disorders [13,14].

Given that Spastic Diplegia form is the most common form with around 65-70 % of the CP children, a review is warranted to assess the effects of NIBS on the lower extremities [15].

This study outlines a protocol for a systematic review evaluating the effectiveness of transcranial Direct Current Stimulation (tDCS) and repetitive Transcranial Magnetic Stimulation (rTMS), in children with spastic cerebral palsy. To the authors' knowledge, no prior systematic reviews have been published that assess the effectiveness of NIBS in enhancing lower extremity function in this population. This review seeks to provide a clear understanding of the various stimulation parameters and the impact of NIBS, addressing whether these interventions lead to changes in motor function and how they influence daily activities and gross motor performance.

## Description of protocol

### Methods

This review protocol follows the recommendations of the Preferred Reporting Items for Systematic Reviews and Meta-Analyses for Protocols (PRISMA-P) reporting guidelines, and the findings will be reported following the PRISMA guidelines [16]. This systematic review is registered on the International Prospective Register of Ongoing Systematic Reviews (PROSPERO) (registration ID CRD42024602482.) [17].

### Selection process

#### Eligibility criteria

Included studies will meet the following criteria: i) full-text randomized controlled trials published in any language, if possible translation; ii) study participants will be children (age <18) diagnosed with Spastic Cerebral Palsy regardless of the Gross Motor Function Classification System (GMFCS); iii) interventions that implement NIBS as a therapeutic tool, limited to rTMS or tDCS; iv) comparison interventions that include different therapies, sham therapies or no therapy; v) outcome measures reflect lower extremity motor function and related, muscle tone and related, functional mobility and related, balance with quantitative scales, step length and velocity as spatiotemporal gait parameters, cortex excitability measured by MEPs^5^ amplitude and quality of life, activity, and/or participation, as indicated by the ICF^6^.

Studies will be excluded if they show the following criteria: i) other sources or research information such as books, theses, conference proceedings, reports, or scientific posters; ii) studies including participants with other conditions such as other neurological deficits, cancer, fractures, or an inflammatory process; iii) treatments consisting of single sessions, peripheral stimulation or focused only on the upper limbs; iv) studies using NIBS for diagnostic means or measures of cortical physiology rather than for therapeutic means; v) studies comparing NIBS protocols (different frequencies, duration, number of pulses, site of stimulation, intensity); vi) studies which do not separately analyse lower extremity outcomes.

### Data collection

Data will be collected on the characteristics of the participants (gender, age, severity) treatment dosage (number of sessions, duration of each treatment session, etc.), description of the intervention and the different comparison treatments. We will not establish any limit in treatment delivery modality (groups, individual, compared intervention).

### Information sources

The following databases will be used for the search: MEDLINE (PubMed), CINAHL Plus, EMBASE, Scopus, ISI WoS and the Cochrane Central Register of Controlled Trials. The search strategy in the PubMed database through the MEDLINE nomenclature and thesauruses is available in supplementary material. The search will be adapted and performed in the other databases mentioned above with the aforementioned terminology and search strategy.

Grey literature will also be searched through the sources recommended by the Cochrane Handbook for systematic reviews of interventions [18], including the Open-Grey database, Google Scholar, the Digitala Vetenskapliga Arkivet, the National Technical Information Service and Trip Database to identify unpublished literature that may meet the inclusion criteria. Besides, a final manual search will be carried out in Google Scholar to check if there is any article that has been omitted or is not included in the databases. If deemed necessary, the author of the included studies will be contacted for additional information. Additionally, two further steps will be implemented to ensure that no relevant results are overlooked. First, a comprehensive search will be conducted in Google Scholar [19], followed by a manual review of the references cited in the most relevant studies.

### Study screening and selection

References will be imported into EndNote v.X9 [20], where duplicates will be removed systematically. After the search, the remaining articles will be imported to Rayyan software for title and abstract screening [21]. Two independent reviewers (CAT and FJVA) will screen titles and abstracts according to the eligibility criteria to determine the suitability of the articles. The full text of potentially eligible studies will then be exported into Covidence [18] for peer review by the same two reviewers. The screening and selection process will be documented using a flow diagram in accordance with the Preferred Reporting Items for Systematic Reviews and Meta-Analyses (PRISMA) guidelines [22]. In cases of disagreement at any stage of the screening process, a third reviewer (FVP) will mediate and resolve conflicts through discussion.

### Data extraction

The data extraction will be peer-reviewed (CAT and FJVA), with a pilot extraction performed initially to ensure consistency. Any uncertainty during the process will be resolved through discussion with a third reviewer (FVP). Data will be extracted from the included studies following the recommendations of the Cochrane Handbook for Systematic Reviews of Interventions [18], using an adapted data extraction form based on the Cochrane Good Practice guidelines [23]. Extracted data will include details on the study population (e.g., number of participants, age, and diagnosis), NIBS interventions and control conditions, outcome assessments, and the statistical effects of the interventions. CAT will input the extracted data into Review Manager software (V.5.3) [24], and a second reviewer (FVP) will verify the accuracy of the data entry.

### Assessing the risk of bias

The risk of bias in the included studies will be assessed using the Cochrane Risk of Bias Assessment tool (Rob2) [25], as recommended by the International Cochrane Collaboration [26]. Two reviewers (CAT and OMN) will independently evaluate the risk of bias for each study, and any disagreements will be resolved through the discussion with a third review author (FVP). Each study will be rated as having a 'low risk,' 'unclear risk,' or 'high risk' of bias based on the reviewers' responses to the tool's questions across different domains and a predefined scoring algorithm.

### Grading of evidence

The Grading of Recommendations, Assessment, Development and Evaluation (GRADE) approach will be employed to assess the certainty of the evidence [27,28]. The GRADEpro software (https://www.gradepro.org) will facilitate this evaluation. Two independent reviewers (CAT and FVP) will conduct the review process, with any discrepancies resolved by a third reviewer (JV). The certainty of the evidence for each outcome will be downgraded based on the following GRADE considerations: limitations in study quality (serious −1 or very serious −2), inconsistency (−1), uncertainty regarding directness (some −1 or major −2), imprecision (−1), and the probability of reporting bias (−1). Conversely, significant effects and a large magnitude of effects (+1) will be considered to potentially upgrade the certainty of evidence [27,28].

### Analysis

#### Descriptive analysis

A narrative synthesis of the findings will be provided from the included studies. The narrative synthesis will be structured according to the following characteristics: i) Type of intervention used as the comparator ii) Type of NIBS applied and parameters iii) Target population characteristics including Gross Motor Function Classification System levels and body involvement (e.g., tetraplegic, diplegic, hemiplegic) iv) Type of outcome measured v) Instrument used for the outcome assessment.

#### Data analysis

Standard Mean difference (SMD) will be re-expressed using a typical scale by multiplying SMD by the standard deviation of the control group at baseline from the most representative study [29,30].

#### Measures of treatment effect

The intervention effect will be analysed using the mean difference (MD) with 95 % confidence intervals (CIs) for continuous outcomes. If the same outcome is measured using different scales, the standardized mean difference (SMD) will be calculated. For dichotomous data, either the risk ratio (RR) or odds ratio (OR) with 95 % CIs will be reported as the effect size. For a better understanding of the results, the absolute and the relative improvement will be calculated. The absolute improvement will be calculated as the mean difference (the improvement in the experimental group minus the improvement in the control group) expressed as a percentage. The relative change will be calculated as the relative difference in the change from baseline (the mean difference divided by the baseline mean of the control group) expressed as a percentage [31,32].

We anticipate a limited scope for meta-analysis due to the variability in outcome measures. However, if the studies included in the review share sufficiently similar characteristics, a meta-analysis will be performed based on the information collected in the data extraction form.

#### Assessment of heterogeneity

We will utilize forest plot analysis and conduct standard χ² tests with a significance level set at α = 0.1 [33]. Additionally, the I² statistic will be employed to detect and quantify the level of heterogeneity among the studies included in our analysis and its impact on the meta-analysis [34,35]. Furthermore, we will thoroughly assess potential factors contributing to this heterogeneity by examining the characteristics of individual studies and subgroups.

#### Data synthesis

Quantitative data synthesis aims to integrate information from various studies to create a cohesive overview [36]. This will be accomplished through a meta-analysis, in which a quantitative summary measure will be calculated based on an evaluation of heterogeneity, study characteristics (including patient demographics, interventions, comparisons, and outcomes), and methodological variations to ensure clinically relevant results. Unless substantial evidence indicates uniform effects across studies, a random-effects model will be employed to consolidate the data [37]. To evaluate the magnitude of the effect size, SMD will be calculated, according to Cohen's categories. It will be considered an SMD >0.2-0.5 as a small effect size, SMD >0.5-0.8 as a medium effect size, and SMD >0. As a large effect size [38]. If at least 10 studies are available, a prediction interval will be provided to estimate the expected range of the true treatment effect in individual studies [39]. Heterogeneity across studies will be assessed using the Q-statistic; however, given its limited power with a small number of studies, the I² statistic will also be calculated to distinguish the proportion of actual variation in effect size attributable to true differences versus sampling error [33]. If a meta-analysis is unfeasible, a narrative synthesis will be adopted following the Cochrane Handbook guidelines [18,40].

#### Subgroup analysis

If enough data is collected, we will conduct a subgroup analysis to assess how different modes of intervention delivery (tDCS and rTMS), and various types of control groups (including other therapeutic interventions and sham control groups) impact the outcomes. This analysis aims to provide a more nuanced understanding of the effects associated with different intervention delivery methods, thereby contributing valuable insights into future research and clinical practice in this field.

#### Sensitivity analysis

If at least 10 studies are obtained, we will conduct a sensitivity analysis to evaluate the robustness and reliability of the conclusions drawn from our meta-analysis [41].

### Ethics and dissemination

Ethical approval is not required for the protocol or the subsequent systematic review. However, the authors will adhere to ethical standards in data management and ensure that the presentation and discussion of findings comply with ethical guidelines at every stage of the research. The authors intend to disseminate the study results through peer-reviewed scientific journals and relevant national and international conferences. They declare that they do not anticipate any modifications to the review methods outlined in this protocol. Should adjustments become necessary, these will be documented, and approval from all reviewers will be required for any amendments. Corresponding changes will also be reflected in the PROSPERO registration record.

## Protocol validation/discussion

Cerebral palsy (CP) is one of the most common causes of motor disability in children [42]. The diagnosis is primarily based on non-progressive motor function and posture disorders, which may change with age. As the child grows, there can be a deterioration in the neuromuscular system and functional capabilities, even though the underlying brain injury remains static [1,7].

The greatest potential for modification occurs during the early stages of central nervous system development, owing to its high capacity for plasticity, which facilitates compensation for various deficiencies. For this reason, rehabilitation for children with cerebral palsy should begin as early as possible. Several physiological mechanisms make non-invasive brain stimulation a groundbreaking therapy [43] particularly through the induction of long-term potentiation (LTP) or long-term depression (LTD)-like protocols [[44], [45], [46], [47]] which are strongly associated with motor learning [45,48,49].

However, the variability in study designs and outcome measures has limited definitive conclusions regarding its impact on functional improvements, and the focus has been largely on upper extremity motor functions. A recent systematic review on the safety and efficacy of NIBS for the upper extremities in children with CP [50], demonstrated that both rTMS and tDCS are safe and feasible for this population. Nevertheless, the effects on upper extremity structure, function, and activity outcomes remain unclear due to the variability in study designs. Notably, none of the studies reported a lack of efficacy, which contrasts sharply with findings in populations with adult neurological diseases [51,52].

This review represents a critical step forward as the first effort to systematically assess the efficacy of NIBS in improving lower extremity motor function in children with spastic CP. Lower extremity function is crucial for mobility, independence, and overall quality of life, yet research in this area has been significantly limited. By consolidating and analysing existing data, this review aims to fill a crucial gap in the literature and provide clearer insights into the potential of NIBS as a therapeutic option for improving lower extremity function.

The results of this systematic review could have significant implications for clinical practice. A better understanding of the effects of NIBS on lower extremity function could inform the development of more targeted rehabilitation protocols, enhance the quality of life for children with CP, and potentially reduce the long-term burden of disability. Additionally, by identifying gaps in the current evidence, this review may guide future research, paving the way for more robust and focused clinical trials that could refine and optimize the use of NIBS for this population.

## Limitations

The authors anticipate challenges and constraints during this research. One limitation is the expected diversity in non-invasive brain stimulation parameters. A second limitation is the variations in scales used to assess outcomes. However, this scenario presents an opportunity to identify and document the underlying causes of such diversity. Despite these potential limitations, the insights gained from this study are expected to enhance our understanding of the relationship between NIBS interventions, their delivery modalities, and their impact on managing this chronic condition.

## CRediT author statement

**Clàudia Arumí-Trujillo:** Conceptualization, Data curation, Investigation, Methodology, Visualization, Writing – original draft, Writing – review & editing, **Francisco José Verdejo-Amengual:** Data curation, Investigation, Methodology, Writing – review & editing, **Oriol Martínez-Navarro:** Data curation, Investigation, Methodology, Writing – review & editing, **Jord J.T. Vink:** Investigation, Methodology, Project administration, Supervision, Validation, Writing – review & editing, **Fran Valenzuela-Pascual:** Investigation, Methodology, Project administration, Supervision, Validation, Writing – review & editing.

## Declaration of competing interest

The authors declare that they have no known competing financial interests or personal relationships that could have appeared to influence the work reported in this paper.

## Data Availability

No data was used for the research described in the article.

## Appendix Supplementary materials

Image, application 1

## Footnotes

5 Motor evoked potential.

6 International Classification of Functioning, Disability and Health.
