# Supplementary material for: The effectiveness of non-invasive brain stimulation in enhancing lower extremity function in children with spastic cerebral palsy: Protocol for a systematic review and meta-analysis
Source: MethodsX. 2024 Dec 31;14:103141. doi: 10.1016/j.mex.2024.103141 (PMC11755019; doi:10.1016/j.mex.2024.103141)
Supplement: Supplementary file 1 [file mmc1.docx]

**Table 1**

| *Search strategy PubMed* |
| --- |
| *((("Cerebral palsy"[Mesh] OR “Cerebral palsy”[tiab] OR ((“Cerebral Pals*”[tiab] OR “Disease”[tiab]) AND (“CP”[tiab] OR “Dystonic-Rigid”[tiab] OR “Distonic”[tiab] OR “Mixed”[tiab] OR “Infantile”[tiab] OR “Rolandic Type”[tiab] OR “Congenital”[tiab] OR “Little”[tiab] OR “Spastic”[tiab] OR “Monoplegic”[tiab] OR “Athetoid”[tiab] OR “Dyskinetic”[tiab] OR “Atonic”[tiab] OR “Hypotonic”[tiab] OR “Diplegic”[tiab] OR “Spastic”[tiab] OR “Quadriplegic”[tiab])))) AND ((“Adolescent”[Mesh] OR “Adolescent*”[tiab] OR “Teen*”[tiab] OR “Young*”[tiab] OR “Youth”[tiab] OR (“Adolescent*”[tiab] AND (“Female*”[tiab] OR “Male*”[tiab]))) OR (“Child”[Mesh] OR “Infant”[Mesh] OR “Pediatric”[tiab] OR “Children”[tiab]))) AND (("Transcranial Magnetic Stimulation"[Mesh] OR “Transcranial Magnetic Stimulation*”[tiab] OR “rTMS”[tiab] OR “r-tms”[tiab] OR “Repetitive Transcranial Magnetic Stimulation”[tiab] OR “EMT”[tiab] OR “non-invasive brain stimulation”[tiab] OR “Direct Current Stimulation”[tiab] OR “neurostimulation methods”[tiab] OR “neurostimulation”[tiab] OR “muscle spasticity”[tiab]) OR ("Transcranial Direct Current Stimulation"[Mesh] OR “Transcranial direct current stimulation”[tiab] OR ((“tDCS*”[tiab] OR “Random Noise Stimulation”[tiab] OR “Alternating Current Stimulation”[tiab] OR “Electrical Stimulation*”[tiab]) AND (“Cathodal Stimulation”[tiab] OR “Transcranial”[tiab] OR “Anodal Stimulation”[tiab] OR “Repetitive Transcranial”[tiab])) OR “tDCS”[tiab] OR “non-invasive brain stimulation”[tiab] OR “Direct Current Stimulation”[tiab] OR “neurostimulation methods”[tiab] OR “neurostimulation”[tiab])) AND ((("Motor Skills Disorders"[Mesh] OR “Motor Skills Disorder*”[tiab] OR (“Disorder”[tiab] AND (“Motor Skill*”[tiab] OR “Developmental Coordination”[tiab]))) OR ("Walking"[Mesh] OR “Walking”[tiab] OR “Ambulation”[tiab] OR “Gait analysis”[tiab]) OR ("Mobility limitation"[Mesh] OR “Mobility Limitation*”[tiab] OR ((“Limitation*”[tiab] OR “Difficult*”[tiab] OR “Walking”[tiab] OR “Walking”[tiab]) AND (“Mobility”[tiab] OR “Ambulation”[tiab] OR “Difficulty”[tiab]))) OR ("Canes"[Mesh] OR “Cane*”[tiab] OR “Walking Stick*”[tiab]) OR ("Wheelchairs"[Mesh] OR “Wheelchair*”[tiab] OR “Wheel Chair*”[tiab]) OR ("Walkers"[Mesh] OR “Walker*”[tiab]) OR (“Gross motor function”[tiab] OR “GMFCS”[tiab])) OR (("Muscle spasticity"[Mesh] OR “Muscle spasticity”[tiab] OR “Spastic”[tiab] OR (“Spasticity”[tiab] AND (“Clasp-Knife”[tiab] OR “Clasp Knife”[tiab]))) OR ("Muscle tonus"[Mesh] OR “Muscle tonus”[tiab] OR (“Tension”[tiab] OR “Tightness”[tiab] AND (“Muscular”[tiab] OR “Muscle”[tiab])))) OR (("Quality of Life"[Mesh] OR “Quality of Life”[tiab] OR (“Quality of Life”[tiab] AND (“Health-Related”[tiab] OR “Health Related”[tiab])) OR “Life Quality”[tiab] OR “HRQOL”[tiab] OR “QoL”[tiab]) OR ("Personal autonomy"[Mesh] OR “Personal autonomy”[tiab] OR “Self Determination”[tiab] OR “Free Will”[tiab]) OR ("Personal Satisfaction"[Mesh] OR “Personal Satisfaction”[tiab] OR “Satisfaction”[tiab] OR "Patient Satisfaction"[Mesh] OR “Patient Satisfaction”[tiab]) OR ("Family Health"[Mesh] OR “Family Health”[tiab] OR "Caregiver Burden"[Mesh] OR “Caregiver Burden”[tiab] OR (“Burden*”[tiab] OR “Exhaustion”[tiab] AND (“Caregiver*”[tiab] OR “Care”[tiab] OR “Care Giving”[tiab])))) OR ("Physical fitness"[Mesh] OR “Physical fitness”[tiab] OR “Functional Gain”[tiab]))* |
